# Night work and BMI: is it related to on-shift napping?

**DOI:** 10.11606/S1518-8787.2017051007094

**Published:** 2017-11-07

**Authors:** Aline Silva-Costa, Rosane Härter Griep, Lúcia Rotenberg

**Affiliations:** I Fundação Oswaldo Cruz. Instituto Oswaldo Cruz. Laboratório de Educação em Ambiente e Saúde. Rio de Janeiro, RJ, Brasil

**Keywords:** Nursing, Team, Shift Work, Sleep Deprivation, Body Mass Index, Occupational Health

## Abstract

On-shift napping can benefit night workers regarding sleep loss, synchronization of circadian rhythms, and alertness. However, few studies on napping can be found in the literature focused on possible health benefits. This cross-sectional study has investigated the role of on-shift napping on the association between night work and BMI in 409 night-shift nursing professionals. The number of working nights and the years of exposure to night work were significantly associated with increased BMI levels among non-nappers, but not among nappers. Results suggest a benefit of napping for weight gain, thus subsidizing occupational health policies on the regulation of on-shift napping among nursing workers.

## INTRODUCTION

On-shift napping has been suggested as a countermeasure to the exposure to night work in relation to psychophysiological, performance, and subjective measures, such as fatigue and alertness^4^. A review paper has examined the potential benefits of strategic napping among pilots, showing that naps not only help to maintain and restore alertness and performance, but they also reduce the accumulated sleep debt and subjective feelings of fatigue^4^. Among nurses, on-shift napping has also been adopted as a strategy to address the negative health consequences of sleep debt related to night work^6^. However, few studies have examined the benefits of napping in relation to health outcomes. Napping during night work has been proposed as a protective factor against self-reported physician diagnosis of hypertension^5^. Moreover, it improves neuroendocrine stress and immune recovery, with a potential prophylactic long-term effect on cardiovascular health^2^.

On-shift napping may compensate sleep loss and contribute to the synchronization of the circadian rhythms among night workers, as it occurs at a proper time from a chronobiological perspective^4^ ^,^ ^7^. Considering that night work has been suggested as a risk factor for obesity or weight gain^3^, we hypothesized that the negative effects of night work on body weight could be attenuated by on-shift napping. The aim of this study has been to investigate the association between night work (number of working nights and years of exposure to night work) and body mass index (BMI), considering on-shift napping.

## METHODS

### Study Population

This cross-sectional study was conducted in a public hospital in Rio de Janeiro, Brazil, with nursing professionals (nursing aides/assistants and registered nurses) who were invited to participate in a face-to-face approach by a team of interviewers. Workers had a schedule of a 12-hour night work (7:00 a.m.–7:00 p.m.) followed by either 36 or 60 hours off. This work scheme allows them to engage in more than one job. Therefore, the definition of the work variables considered all jobs in which the workers were engaged.

The initial sample comprised 1,224 workers of whom 583 were current night workers. The group was reduced to 445 workers after excluding those who had worked at night for less than one year (n = 32) and the men (n = 106). After the exclusion of data corresponding to workers who had missing data or who had differences regarding on-shift napping at different workplaces (n = 36), the final sample comprised 409 female nursing professionals with at least one year of night shift work.

The study was approved by the Research and Ethics Committees of the hospital (Protocol 000.472, in June 12, 2012). All individuals who participated in the study provided written informed consent.

### Variables

Body mass index (BMI) was calculated by dividing the participants’ weight (in kilograms) by the square of their height (in meters). Weight measurements were performed using a digital scale (Tanita^®^, model Solar HS-301 which can accurately measure up to 150 kg), and height measurements were obtained using a portable stadiometer (Alturexata^®^ with scale increments of 0.1 cm). The BMI was analyzed as a continuous variable.

In relation to number of working nights, we have considered as night workers those who reported working night shifts (7:00 p.m.–7:00 a.m.) regularly (at least once a week or four times a month) in nursing care. Current night workers were invited to recall: “What nights did you work over the last two weeks, considering the total number of working nights in all jobs?”

To estimate the time of exposure to night work, current night workers were asked: “How long have you been working at night in nursing, here or in another place?”

Regarding on-shift napping, current night workers were asked: “In relation to your hours of sleep or rest during the night shift, would you say that most of the time you (1) just rest (can’t sleep), (2) sleep, or (3) do not sleep nor rest”. Nursing workers who answered “2” were classified as nappers, and those who answered “1” or “3” were classified as non-nappers.

Besides the above mentioned data, the instrument of data collection also provided also provided information on age (years), education (high school or university), smoke status (never, former and current smoker), alcohol consumption (yes or no), duration of leisure physical activity (minutes/week), self-reported weight at 20 years (kg), sleep duration (hours/day), and work hours (hours/week).

### Data Analysis

A gamma regression model (GLM) with an identity link function was used to test the association between the number of working nights and the years of exposure to night work and BMI, considering on-shift napping. The option for the gamma regression was based on the continuous, strictly positive, and skewed nature of the dependent variable (BMI). The link identity was chosen because it provides an easy and direct interpretation of the model coefficients. The coefficients (β-values) and 95% confidence intervals (95%CI) were estimated from multiple models, beginning with an unadjusted model (Model 1), and then adjusting for the potential confounders (Model 2: adjusted for age, education, leisure physical activity, smoke status, sleep duration, work hours, and weight at 20 years). Analyses were performed using the software R, version 2.15.

## RESULTS

From the study sample, 193 workers (47.2%) were nappers. The average age of participants was 43.4 years (SD = 11.4) and 40.2 years (SD = 10.3) for non-nappers and nappers, respectively. The mean number of working nights per fortnight was four nights. The mean BMI were 27.9 (SD = 5.1) and 27.6 (SD = 5.5) for non-nappers and nappers, respectively. Data on nappers showed that 15.5% and 84.5% of the sample reported on-shift naps of up to 120 minutes and from 121 to 180 minutes, respectively. Analysis considering nap duration did not show differences, so nappers were analyzed as a single group.

Among non-nappers, number of working nights and years of night work were significantly associated with increased BMI levels (β-value = 0.364 and β-value = 0.092, respectively, after adjusting for the potential confounders). However, in relation to the group of night workers who usually nap during the night shift, the association between night work and BMI was not statistically significant (Table).

**Table:** Coefficients (β-values) and 95% confidence intervals (95%CI) for the associations between night shift – working nights and years of night work – and body mass index according to on-shift napping.

Variable	BMI			

	Model 1^a^		Model 2^b^	
	
	β-values	95%CI	β-values	95%CI
Non-nappers				
Number of working nights	0.427^d^	0.002–0.854	0.364^d^	0.002–0.749
Years of night work	0.172^c^	0.096–0.249	0.092^c^	0.011–0.173
Nappers				
Number of working nights	0.065	-0.462–0.613	0.120	-0.341–0.586
Years of night work	0.149^c^	0.046–0.256	0.092	-0.018–0.203
Nappers + Non-nappers				
Number of working nights	0.220	-0.101–0.544	0.202	0.074–0.479
Years of night work	0.176^c^	0.116–0.237	0.102^d^	0.039–0.165

^a^ Model 1: unadjusted model.

^b^ Model 2: adjusted for age, education, physical activity, smoke status, weight at 20 years, sleep duration, and work hours.

^c^ p < 0.001

^d^ p < 0.05

## DISCUSSION

The fact that significant associations between night work and increased BMI were restricted to workers who did not nap suggests a benefit of on-shift napping in relation to body weight. This result is in line with those suggesting on-shift napping as a countermeasure to the negative effects of night work in relation to physiological measures^2^ and self-reported physician diagnosis of hypertension^5^.

Circadian misalignment and sleep loss resulting from the exposure to night work are among the mechanisms underlying obesity in night workers. Exposure to night work, with a decrease or cessation in melatonin secretion, also alters the temporal organization of metabolic functions, because the release of melatonin follows a daily pattern that is important in maintaining the circadian synchronization between the activity and rest rhythms^1^. Therefore, the role of on-shift napping on BMI may also involve a regulation in the melatonin level, which is altered in night workers. On-shift napping may contribute to the increase in the total sleep duration and also to the synchronization of the circadian rhythms of night workers, since naps taken when the circadian drive to sleep is at its highest have shorter sleep onset latencies than naps taken at other times during the day^4^. Also, in another study with nursing professionals, the efficiency of this short sleep during night work was similar to the one observed in the nighttime sleep at home during rest days^7^, although the duration and quality of these naps are usually not as adequate as the full sleep at home^4^ ^,^ ^7^. Moreover, agreement about the best time to nap and the duration of on-shift napping among night workers is still a challenge.

This study had some limitations. Possibly, the timing of the on-shift napping – from 0:00 a.m. to 3:00 a.m. or from 3:00 a.m. to 6:00 a.m.^7^ –, which was not obtained in this study, could have influenced the results. For example, a previous study has shown that the efficiency of nighttime sleep at work is higher among those napping after 3:00 a.m., and it may be related to the levels of melatonin: maximum level of melatonin release and lower core temperature are observed approximately between 3:00 a.m. and 4:00 a.m.^7^. Although napping depends on low workload, studies on nursing workers of public hospitals suggest that the practice of allowing workers to sleep during night shifts is common, with low percentages of workers reporting otherwise^7^. In addition, the analyses were adjusted for potential confounding factors, but we cannot rule out the possible occurrence of residual effects or uncontrolled variables. Although the cross-sectional nature of the study does not allow us to establish the association directionality, these results were interpreted based on the literature concerning the negative effects of night shift work and sleep loss on weight gain. Finally, this study has an important strength regarding the real-life approach, which is especially valuable for the investigation of the benefits of on-shift naps^5^.

In summary, the results from this study, which is in agreement with other research showing the positive effects of napping during night work in relation to recovery from work^6^ and hypertension^5^, suggest a potential benefit of napping among nursing workers. This discussion can support occupational health policies for the regulation of naps as it strengthens the evidence from the literature on the importance of on-shift napping for the health of night workers.
